# Impaired sleep quality is associated with concurrent elevations in inflammatory markers: are post-menopausal women at greater risk?

**DOI:** 10.1186/s13293-019-0250-x

**Published:** 2019-07-08

**Authors:** Bianca D’Antono, Véronique Bouchard

**Affiliations:** 1 Research Center, Montreal Heart Institute, 5000 Belanger Street, Montreal, Quebec, H1T 1C8 Canada; 2 Psychology Department, Université de Montréal, Montreal, Quebec, Canada

**Keywords:** Sleep, C-reactive protein, TNF-α, IL-6, Sex, Menopause

## Abstract

**Abstract:**

**Background:**

Chronic inflammation and impaired sleep increase the risk for cardiovascular disease. Menopausal women may be particularly at risk as a result of impaired sleep. The objective of the current investigation was to assess the relationship between poor sleep and C-reactive protein (CRP), interleukin-6 (IL-6), tumor necrosis factor alpha (TNF-α), and myeloperoxidase (MPO) in healthy non- and postmenopausal women and men.

**Methods:**

A fasting blood draw was obtained from 122 healthy men and women (31 were postmenopausal). Higher scores on the Pittsburgh Sleep Quality Index (PSQI) were used to define poor sleep. Given the sample size and healthy nature of the sample, hierarchical linear regression analyses were performed on a composite inflammatory score involving CRP, IL-6, and TNF-α. Sex/menopausal group and PSQI were entered as predictors, and the interaction of the group by PSQI was entered stepwise. Analyses on MPO were performed separately.

**Results:**

Sleep quality was associated with higher inflammatory activity (*β* = 0.272, *P* = 0.003), which remained significant (*P* = 0.046) after controlling for age, waist circumference, exercise times per week, and depressive symptoms*.* While in the same direction, sleep quality was not significantly associated with MPO. Dichotomizing sleep quality led to similar results.

**Conclusion:**

Impaired sleep quality is independently associated with greater inflammation in healthy adult men and women. Despite an overall less favorable metabolic and inflammatory profile in postmenopausal women, impaired sleep did not emerge as differentially related to inflammatory activity in this group.

## Background

Sleep impairment, defined as sleep of short duration, presence of insomnia symptoms, or non-restorative sleep, is associated with a number of health outcomes in cross-sectional and prospective epidemiological studies, including increased risk for cardiovascular (CV) morbidity and mortality [1–10]. Sleep impairment may contribute to CV disease (CVD) through its association or impact on other intermediary risk factors for CVD, including increased body weight [11, 12], hypertension [8, 13, 14], changes in glucose metabolism/diabetes, and insulin resistance [15]. Changes in inflammation as a result of sleep disturbance may also be involved [16–22].

Proinflammatory cytokines, such as interleukin-6 (IL-6) and tumor necrosis factor-alpha (TNF- α) play a central role in the formation and progression of atherosclerotic plaque in the arterial wall [23–25]. Inflammatory processes involving TNF-α, a major regulator of the cytokine cascade involving both pro and anti-inflammatory mediators [25], trigger the induction of IL-6 and promote the production of acute-phase proteins such as C-reactive protein (CRP) [23, 24]. Chronic low-grade inflammation, as represented by C-reactive protein (CRP), is an emerging risk factor for the development of atherosclerosis and its complications [26, 27] and predicts CAD and mortality independently of traditional risk factors [14, 28–31]. For this reason, CRP is increasingly measured as part of the individual risk evaluation for heart disease [32, 33].

Experimental studies involving short-term sleep deprivation in healthy individuals suggest that sleep disturbance leads to acute changes in inflammatory (including TNF-α, IL-6, CRP), metabolic, and other responses that could contribute to cardiovascular disease processes [34]. While contradictory data exists [16], CRP was shown to increase twofold following short-term experimental sleep restriction to 4 h per night and fourfold after 10 days of partial sleep deprivation [35]. Total sleep deprivation for 3 days led to clinically significant elevations in CRP in healthy individuals. Fragmented sleep because of obstructive sleep apnea is similarly associated with significantly higher values of TNF-α, IL-6, and CRP as compared to preserved sleep [34, 36–41]. The quality (rather than or in addition to quantity) of sleep is also related to elevations in inflammatory markers, including CRP and IL-6 [16]. For example, in community-dwelling adults, excessive daytime sleepiness [42] and poor sleep [18, 43–46] were associated with higher circulating levels of IL-6 compared to individuals with better sleep. Sleep disturbance was also associated with heightened systemic inflammation in a general population over a 5-year follow-up [21].

Sample characteristics may influence the relationship between poor sleep and CV outcomes. Indeed, while men are at increased risk of CVD, some limited data suggests that women may be at higher risk for sleep-related CV consequences than men [3, 47–49]. Similarly, while the consequences of poor sleep on metabolic abnormalities have been reported in both sexes [15], its impact on inflammatory mechanisms and their downstream marker CRP may be sex-specific appearing only or to a greater extent in women [16, 17, 50, 51]. For instance, while inconsistent results have been obtained among younger individuals [52], data from the phase 3 Whitehall II cohort (4642 middle-aged participants) found that women sleeping less than 5 h a day on average had CRP values that were 42% higher than those sleeping 7 h, after adjusting for BMI, marital status, SBP, and triglyceride levels [17]. Since CRP predicts more cardiovascular events in women compared to men [53], such a sex-specific effect of sleep impairment on inflammatory activity may be all the more damaging.

A particularly at-risk group of women for such adverse effects may be postmenopausal women. Menopause is an important CV risk factor both for the negative effect of ovarian hormone deprivation on CV function and for the consequent worsening of risk factors [54, 55]. These include changes in body fat distribution from a gynoid to an android pattern, reduced glucose tolerance, increased blood pressure, decreased blood pressure dipping, alterations to the lipid profile, and changes in proinflammatory activity [17, 54–57]. Sleep impairment also appears to increase with menopause [58–67]. Sleep complaints are reported by 25–60 % of all women transitioning into menopause [63–65], as compared to about 12–15% of the general population [68, 69]. In the Survey of Women’s Health Across the Nation, postmenopausal women reported a 1.6–3.4-fold greater occurrence of sleep disturbances, including symptoms of insomnia and sleep apnea, compared to premenopausal women [58]. Importantly, Prinz et al. [70] examined IL-1 beta concentrations in healthy seniors and found higher circulating levels in postmenopausal women with impaired sleep quality. This was not observed in men. To our knowledge, it has not yet been investigated whether the association between inflammatory activity and poor sleep differs as a function of menopausal status in women.

Given the impoverishment of sleep quantity and quality observed over the past decades and with age [34], understanding the importance of sleep to health in both men and women, particularly at-risk women, is essential. In this study, we assessed CRP, IL-6, TNF-α, and myeloperoxidase (MPO, a marker of lipid oxidation) in healthy premenopausal and postmenopausal women, as well as in men presenting varying sleep quality. We hypothesized that poor sleep would be independently and specifically associated with higher values of inflammatory activity in women, particularly postmenopausal women.

## Methods

This study reports on the cross-sectional association between poor sleep quality and inflammatory activity in healthy premenopausal and postmenopausal women and men.

### Participants

The study sample consisted of 132 healthy men and women enrolled in a prospective investigation 3 years earlier. They had originally been recruited through advertisements in newspapers and community centers within the greater Montreal area. Of the original 199 participants recruited between 2005 and 2007, we were able to reach 184 participants, 143 of whom agreed to participate. Thirty-five individuals refused to participate because of lack of interest, incompatible schedules, or perception of testing as too demanding. An additional 6 participants were excluded for medical reasons (cancer, pregnancy, post-partum, and sleep apnea). Those who agreed to participate in the follow-up were slightly older (*P* = 0.044) and smoked less (*P* = 0.013) compared to those who declined. Of the 143 individuals who participated in the follow-up study, 132 had complete data required for analysis in this study.

At study entry, participants met the following eligibility criteria: (a) no utilization of mental health services within the past year; (b) no medication known to affect cardiovascular, immune, or neuro-endocrine functions; (c) no previous diagnosis of sleep apnea; (d) no oral contraceptive and hormone replacement therapy; and (e) no learning or cognitive disabilities capable of impairing the ability to complete questionnaires or understand instructions. To ensure a broad age distribution, participants were initially selected to provide approximately three equal age groups (18–34 years; 35–44 years; 45–65 years). Women were over-sampled to include a sufficient number of postmenopausal women. Menopausal status at follow-up was indicated by the (1) absence of a menstrual cycle for 12 months or more prior to testing that was not due to any medical condition (e.g., hysterectomy, anorexia), as well as (2) follicular stimulating hormone (FSH) levels between 23.0–116.3 U/L and estradiol levels between 0–198 pmol/L. Of the potential postmenopausal women at follow-up, 10 women were excluded from further analysis as they met only one or the other of these requirements. Thus, the final sample used for the remaining analyses consisted of 53 men, 38 premenopausal women, and 31 postmenopausal women (total *N* = 122).

All subjects signed the informed consent form which was approved by the Research and Ethics Board of the Montreal Heart Institute. Participants were paid $250 for participation in this study.

### Procedures at follow-up

Eligible participants were scheduled for a laboratory appointment at the Montreal Heart Institute. To control for circadian rhythms in physiological activity, laboratory visits were scheduled for 8:00 am on weekdays. Participants were asked to abstain from eating, drinking (other than water), and smoking as well as refrain from strenuous physical activity for 12 h prior to testing. They were also asked to refrain from alcohol or drug use (including aspirin and non-steroidal anti-inflammatory medications) during the 24-h period preceding the appointment. Participants who did not adhere to these instructions on the day of testing or who presented physical symptoms (such as cough, cold, or headache) were sent home and a new appointment was scheduled to limit biasing inflammatory values as a result of acute infections. Post hoc exclusion of participants with hsCRP > 10 was planned for the same reason.

During the laboratory session, participants were interviewed as to sociodemographic, health behavior, and medical history. Anthropometric measurements (including waist circumference) were obtained. A blood sample was taken after 10 min of rest in a semi-reclined position. Subjects also completed questionnaires on sleep and depressive symptoms. Following laboratory testing, subjects underwent a 24-h ambulatory blood pressure (BP) monitoring using Spacelab Ambulatory Blood Pressure Units (Model 90207-30; Redmond, WA).

### Measures

Data on sex, age, ethnicity, waist circumference, height, body mass index (BMI), years of schooling, personal/family income, marital status, alcohol/tobacco consumption, and physical activity were collected.

*The Pittsburgh Sleep Quality Index (PSQI)* [71] is a validated 19-item self-report questionnaire assessing sleep complaints and overall sleep quality over the previous month, with a higher global PSQI score indicating worse sleep quality. It demonstrates an internal consistency of 0.83 and a ± 1-month test-retest reliability of 0.85. A global PSQI score > 5 reflects poor sleep quality, differentiating between clinical determinations of good and poor sleepers with a diagnostic sensitivity of 89.6% and specificity of 86.5%. Good to excellent psychometric properties were similarly shown in individuals with primary insomnia [72].

*The Beck Depression Inventory–II (BDI-II)* [73] is a 21-item scale measuring the behavioral manifestations and severity of depressive symptoms. It has excellent test-retest reliability (*r* = 0.80–0.90) as well as good internal consistency (*α* = 0.73–0.95). Considerable evidence attests to the importance of depression in coronary artery disease [74] and inflammation [75].

Blood for CRP measurement was collected in plain tubes and analyzed thereafter using the Siemens (formerly Dade Behring) CardioPhase hsCRP assay (Siemens Healthcare Diagnostics Products GmbH, Marburg, Germany). The minimal detectable hsCRP concentration was 0.18 mg/L.

*IL-6* was measured from serum using the R&D Systems Quantikine High Sensitivity Il-6 ELISA assay (Cat. No. HS600B, R&D Systems, Minneapolis, USA). The smaller standard (0.156 ng/L) was used as the sensitivity level.

*TNF-α* was obtained from serum, using the R&D Systems Quantikine High Sensitivity TNF-α ELISA assay (Cat. No. HSTA00D, R&D Systems, Minneapolis, USA). We used the smaller standard (0.5 ng/L) as the sensitivity level.

*MPO* is a hemoprotein secreted during inflammation and an indicator of oxidized lipids [76]. It was measured from plasma using the ALPCO Diagnostics Myeloperoxidase (MPO) ELISA assay (Revised version, Cat. No. 30-6631A, ALPCO Diagnostics, Salem, NH, USA). We used the smaller standard (1.9 μg/L) as the sensitivity level.

Blood samples were also analyzed for lipids, glucose, and insulin at the Montreal Heart Institute. These determinations were made using respective reagent Flex on the multianalyzer Dimension RxL Max (Dade Behring Diagnostics, Marburg, Germany) with heparinized plasma, as simultaneously as possible following the blood draw. Insulin was measured by electrochemiluminescence (ECL) immunoassay using the Roche Insulin assay (Roche Diagnostics GmbH, Mannheim, Germany) on the Cobas e601 analyzer (Roche Diagnostics).

BP measures were obtained every 20 min in the daytime and every hour from 22:00 to 06:00 h. Average nighttime and daytime values of SBP and DBP were calculated. At least 70% of recorded BP readings were satisfactory for each period, as per recommendations of the European Society of Hypertension and the European Society of Cardiology Task Force [77]. For nighttime, this represented a minimum of six out of eight hourly measures.

### Analyses

Descriptive statistics were used to characterize the sample.

A composite score was used to reflect the overall inflammatory activity. The individual inflammatory markers were each standardized (to a mean of 0 and a standard deviation of 1) and then summed (*z*(CRP) + *z*(IL-6) + *z*(TNF-α)), as per research involving the metabolic syndrome construct [78–81]. Potential covariates were based on the literature and included demographic, behavioral, metabolic, and hemodynamic variables. Their correlations with PSQI, MPO, and the composite inflammation score are shown in the “Results” section if they correlated at *P* < 0.15. However, given the sample size, number of covariates, and correlations among them, a preliminary stepwise regression was performed to reduce the number of covariates. The composite inflammation score was entered as a dependent variable and the potential covariates as predictors. For these preliminary analyses, the *P* value for entry was 0.15. This was repeated for MPO. The covariates that were retained for the composite inflammation score were age, waist circumference, exercise times, and BDI-II scores, explaining 23.5% of the variance. HDL-C (and age) were retained for MPO, explaining only 2.9% of the variance.

The primary endpoints (composite inflammation score, MPO) were assessed as a function of sleep quality (continuous variable) and sex/menopausal group membership (men, non-menopausal women, menopausal women) via a hierarchical regression. Group membership and sleep quality were entered in Block 1 while the interaction between sleep quality and group membership was entered stepwise in Block 2. Analyses were repeated with covariates entered in Block 1, predictors in Block 2, and the interaction term entered stepwise in Block 3.

To examine the association of a more clinically meaningful level of sleep disturbance and inflammatory activity, ANOVAs were performed with the categorical PSQI (> 5 representing the poor sleepers) and sex/menopausal membership as independent variables. This was repeated with the covariates as above.

A two-tailed *P* value < 0.05 was considered statistically significant for these analyses.

## Results

### Descriptive statistics

Baseline characteristics of participants are presented in Table 1. The sample had a mean age of 45.1 ± 11.4 years and had completed a mean of 16 ± 2.8 years of schooling. Twelve percent of the sample were smokers. Subjects were relatively fit, with more than 58% exercising at least once a week. Fifty-five individuals reported poor sleep quality based on a PSQI > 5: nearly half of men and postmenopausal women and 40% of premenopausal women.

**Table 1:** Sample characteristics

Variables	Men (*n* = 53)	Premenopausal women (*n* = 38)	Postmenopausal women (*n* = 31)
Age (years)***	44.5 ± 11.3	37.8 ± 9.3	55.3 ± 4.5
Years of schooling	16.1 ± 3.1	16.1 ± 2.5	15.9 ± 2.6
Family income^1^			
< 30,000$ CDN	11 (21.6%)	6 (15.8%)	8 (25.8%)
30,000–59,999$	19 (37.3%)	15 (39.5%)	12 (38.7%)
≥ 60,000$	21 (41.2%)	17 (44.7%)	11 (35.5%)
Health habits			
Smoking, *n* (%)	8 (15%)	6 (16%)	1 (3%)
Exercise (yes/no)^+^	38 (72%)	22 (58%)	26 (84%)
Exercise (hours/week)*	4.3 ± 4.6	2.2 ± 2.8	4.1 ± 2.7
Metabolic activity			
Body mass index (kg/m^2^)	25.4 ± 3.8	24.9 ± 5.2	25.4 ± 4.0
Waist circumference (cm)*	90.2 ± 9.9	84.6 ± 13.0	83.8 ± 11.2
LDL-cholesterol (mmol/L)**	3.0 ± 0.9	2.7 ± 0.6	3.4 ± 0.8
HDL-cholesterol (mmol/L)***	1.2 ± 0.3	1.4 ± 0.3	1.5 ± 0.3
Fasting glucose (mmol/L)**	5.2 ± 0.6	4.9 ± 0.4	5.4 ± 0.8
Fasting insulin (pmol/L)	47.9 ± 30.5	43.3 ± 21.2	48.4 ± 34.8
Ambulatory BP			
Day SBP (mmHg)**	120.1 ± 8.2	113.5 ± 9.3	116.5 ± 9.5
Day DBP (mmHg)**	74.5 ± 6.4	69.9 ± 7.4	73.1 ± 8.0
Night SBP (mmHg)	107.8 ± 10.5	103.7 ± 12.2	103.7 ± 10.4
Night DBP (mmHg)	62.5 ± 8.3	59.7 ± 8.6	62.1 ± 8.7
Inflammatory markers			
CRP (mg/L)	1.1 ± 1.1	1.5 ± 2.1	1.9 ± 2.2
TNF-α (ng/L)^+^	1.3 ± 0.4	1.2 ± 0.9	1.1 ± 0.3
IL-6 (ng/L)^+^	1.1 ± 0.9	0.9 ± 0.5	1.2 ± 0.7
MPO (μg/L)	53.6 ± 17.7	53.6 ± 14.1	53.6 ± 16.8
Questionnaires			
BDI-II score	6.3 ± 7.9	7.7 ± 8.0	6.6 ± 7.5
PSQI overall score	5.6 ± 2.9	4.8 ± 2.9	6.5 ± 4.1
PSQI > 5, *n* (%)	25 (47%)	15 (40%)	15 (48%)

*CRP* C-reactive protein, *TNF-α* tumor necrosis factor-alpha, *IL-6* interleukin-6, *MPO* myeloperoxidase, *SBP* systolic blood pressure, *DBP* diastolic blood pressure, *LDL* low density lipoprotein, *HDL* high density lipoprotein, *BDI-II* Beck Depression Inventory-II, *PSQI* Pittsburgh Sleep Quality Index

^1^*N* = 120

*P < 0.05

**P < 0.01

***P < 0.001

^+^*P* < 0.07

Groups differed significantly from each other with respect to age. Men had significantly greater waist circumference and lower HDL compared to women. They also had higher daytime BP and glucose compared to premenopausal women. Postmenopausal women exhibited significantly higher glucose and LDL levels compared with premenopausal women, with similar trends for daytime BP and HDL.

### Preliminary analyses

Univariate correlations between potential covariates and inflammatory markers or the PSQI total score for the entire sample are shown in Table 2.

**Table 2:** Bivariate correlations between sleep quality, inflammatory activity, and potential covariates

	PSQI total	Composite inflammation	MPO
Age	0.07	0.16	− 0.01
No. of exercise times	− 0.03	− 0.19*	− 0.02
Smoker	0.18*	0.05	0.15
BMI	0.13	0.39***	0.12
WC	0.14	0.39***	0.13
HDL-C	− 0.07	− 0.13	− 0.17
Glucose	0.22*	0.16	0.14
Insulin	0.12	0.29***	0.10
SBP_daytime_	0.19*	0.04	− 0.11
DBP_daytime_	0.12	− 0.06	− 0.07
SBP_nighttime_	0.09	0.14	− 0.06
DBP_nighttime_	0.04	0.07	− 0.07
BDI-II	0.46***	0.17	0.13
BDI-II (no sleep items)	0.44***	0.16	0.12
PSQI-total	–	0.28**	0.11

The composite inflammation score reflects the sum of standardized values for CRP, IL-6, and TNF-α, specifically *z*(CRP) + *z*(IL-6) + z(TNF-α)

*CRP* C-reactive protein, *TNF-α* tumor necrosis factor-alpha, *IL-6* interleukin-6, *MPO* myeloperoxidase, *SBP* systolic blood pressure, *DBP* diastolic blood pressure, *HDL* high density lipoprotein, *BDI-II* Beck Depression Inventory-II, *PSQI* Pittsburgh Sleep Quality Index

*P < 0.05

**P < 0.01

***P < 0.001

Inflammatory activity was significantly correlated with demographic, behavioral/psychological, metabolic, and/or hemodynamic variables as per the literature.

Impaired sleep quality was associated with smoking status, as well as with significantly higher glucose and daytime SBP values and with more symptoms of depression. It was also associated with significantly higher values of CRP (*r* = 0.31, *P* < 0.001), IL-6 (*r* = 0.20, *P* < 0.05), TNF-α (*r* = 0.19, *P* < 0.05), and the composite inflammation score (*r* = 0.28, *P* < 0.01)

### Multivariate analyses involving continuous PSQI values

#### Composite inflammation score

Only the main effect of sleep quality emerged significant (*β* = 0.272, *t* = 3.068, *P* = 0.003, *r*_partial_ = 0.271) for an overall model (*F*(2,118) = 4.934, *P* = 0.009, *R*^2^ = 0.077, *R*^2^_adj_ = 0.062) that explained 7.7% of the variance in inflammatory activity. Controlling for age, waist circumference, exercise times/week, and BDI-II scores, sleep quality remained significant (*β* = 0.186, *t* = 2.014, *P* = 0.046) for an overall model that explained 26% of the variance (*F*(= 6.758, *P* < 0.001, *R*^2^ = 0.262, *R*^2^_adj_ = 0.224). Effects involving sex/menopausal group membership were not significant.

#### MPO

No significant main or interaction effect of sleep quality or sex/menopausal group membership emerged. Controlling for HDL and age did not alter this.

### Multivariate analyses involving categorical PSQI values

#### Composite inflammation score

**Fig. 1:**
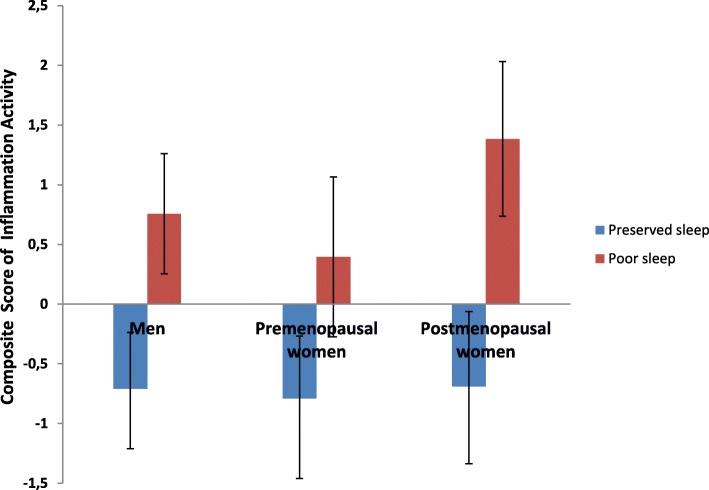
Mean composite inflammatory activity (± SE) as a function of sleep quality and sex/menopausal group membership. The individual inflammatory markers were each standardized (to a mean of 0 and a standard deviation of 1) and then summed (***z***(CRP) + ***z***(IL-6) + ***z***(TNF-α)) to create a composite inflammatory activity score

The ANOVA revealed a significant main effect of sleep quality (*F*(1,115) = 9.480, *P* = 0.003). Individuals reporting impaired sleep showed significantly higher inflammatory activity compared to intact sleepers (0.625 ± SE (0.287) vs. − 0.559 ± SE (0.256)) (see Fig. 1). No main effect or interaction involving sex/menopausal group membership emerged. In the ANCOVA controlling for age, BDI-II, exercise, and waist circumference, the main effect of sleep quality remained significant (*F* = 4.605, *P* = 0.034).

#### MPO

The ANOVA revealed a significant main effect of sleep quality (*F*(1,116) = 3.949, *P* = 0.049). Individuals reporting impaired sleep showed significantly higher MPO values compared to intact sleepers (57.0 ± SE (2.342) vs. 50.7 ± SE (2.118)). No main effect or interaction involving sex/menopausal group membership emerged. The effect became a trend when controlling for HDL-C and age (*F* = 3.568, *P* = 0.061).

### Post hoc analyses

To examine to what extent sleep quality was associated with clinically meaningful values of CRP, i.e., ≥ 3 mg/L, Pearson’s chi-square analyses were performed as a function of sleep quality category separately for men and women with and without menopause. Clinical elevations in CRP were detected in 5 men, 5 premenopausal women, and 6 postmenopausal women. The proportion of individuals with clinical elevations in CRP values was not different between subjects with PSQI ≤ 5 and PSQI > 5 among men (7% and 12%, respectively, *P* = 0.55) and premenopausal women (9% and 20%, *P* = 0.31), whereas 33% of postmenopausal women with PSQI > 5 had CRP ≥ 3 mg/L versus 6% of those with PSQI ≤ 5 (Pearson’s chi-square = 3.6, *P* < 0.056).

## Discussion

The main finding of this study is that poor sleep quality is associated with greater inflammation in healthy adult men and women, independently of age, exercise level, obesity, or symptoms of depression. Only limited evidence existed to suggest that poor sleep was more greatly associated with inflammation in postmenopausal women. More specifically, postmenopausal women experiencing poor sleep quality were more likely to have clinical elevations in inflammation compared to postmenopausal women with preserved sleep. This was not the case for men or premenopausal women.

Sleep loss and sleep disorders have been previously reported to be associated with a proinflammatory state [16–22, 34–46, 82]. Experimentally induced total or partial sleep deprivation has been shown to increase circulating markers of inflammation [34, 35], though inconsistent results have also been obtained [16]. Population-based studies investigating the relationship between poor sleep and inflammation have produced contradictory results [17, 50, 83, 84] that may have been driven, in part by sample characteristics. For instance, a study pooling men and women showed no association between poor sleep and CRP [83]. On the other hand, several studies using sex-based analyses showed this link only, or to a greater extent, in women [16, 17, 50]. For example, in a study implicating 210 non-smoking healthy and unmedicated adult men and women, poor sleep quality was associated with higher values of CRP and IL-6 in women only, after adjustment for age, BMI, and symptoms of depression [50]. The impact of menopausal status was not examined in that study, however. In contrast, in a large Norwegian epidemiological study, self-reported sleep disturbances (assessed via only three questions) were significantly but only very modestly associated with CRP levels in men (Spearman’s *r* ranged 0–0.06 as a function of sleep item), but not in women (Spearman’s *r* ranged − 0.02–0.05) [85]. Associations were no longer significant in men after controlling for cardiovascular risk factors and psychological distress. Menopausal status was not examined in that study. Our investigation extends prior research by showing that the effect of sleep impairment on inflammation is observable in both healthy men and women of varying age, independently of CV risk factors. While it needs to be replicated in a larger sample, sleep impairment may be particularly worrisome, as evidenced by more clinical elevations in CRP in women after menopause. It is also possible that the absence of statistically significant differences in sleep-related inflammatory activity in postmenopausal vs. premenopausal women may reflect the inclusion of perimenopausal women within the premenopausal group. Women in the perimenopausal phase have been shown to suffer from sleep disorders to a greater extent compared with premenopausal women [58, 66, 67], though the impact of this on levels of inflammatory markers has not been examined.

Various factors have been hypothesized to confound the relationship between poor sleep and inflammation. Body mass index, visceral obesity, insulin resistance, and HDL and LDL cholesterol have been reported to be important correlates of CRP and other inflammatory markers [85, 86]. It was certainly the case in this study as well. BP was only marginally associated with poorer sleep (daytime SBP) or elevations in inflammatory markers (nighttime SBP). However, findings remained significant after controlling for obesity, and when this was controlled for, other metabolic parameters no longer significantly contributed to the prediction of inflammatory activity. Depression has also been associated with increased inflammatory processes [75, 87, 88], although the association appears greater among middle-aged (compared to younger) women that are not on hormone replacement therapy [89]. In the current study, while poor sleep was associated with significantly higher depression scores, depression did not explain the relationship between higher inflammatory activity and poor sleep. These results concur with those of Suarez [50] and suggest that depression does not mediate the relationship between poor sleep and inflammation. This said, it is unclear to what extent metabolic parameters (such as obesity) and psychological distress should be controlled for in analyses examining the association of sleep with medical outcomes, as these may be outcomes of poor sleep, rather than confounders per se. Controlling for them may remove part of the variance in inflammatory activity that is in fact attributable to poor sleep.

At this time, the pathophysiological mechanisms responsible for the proinflammatory state in our participants with poor sleep are unknown. Changes in metabolic profile may be implicated, as evidenced by reductions in the significance of associations between sleep and inflammatory markers when controlling for metabolic variables in this and other (e.g., [85]) investigations. Both estrogen and testosterone have been shown to have anti-inflammatory properties acting at genomic and non-genomic levels [90]. The nuclear factor kB (NF-kB) transcription control pathway is a key process in the coordination of the body’s response to stressful situations, infection, and inflammation and controls cellular expression of proinflammatory genes [91]. Irwin and colleagues [92] investigated the effect of partial sleep deprivation for one night on morning NF-kB in a small cohort of middle-aged and older men and women (51 ± 12 years old) and found morning NF-kB to be significantly increased in women but not in men after partial sleep deprivation. Given the age of participants, a large number of women in that cohort were presumably menopausal. No data are currently available for the effects of partial sleep deprivation on morning NF-kB in premenopausal women as compared to postmenopausal women. Nonetheless, it is hypothesized that men and premenopausal women are protected from the harmful effects of poor sleep due to testosterone and estrogen, respectively. Consequently, in the postmenopausal state, with a reduction in circulating estrogen levels, there might be a subsequent increase in inflammation in response to sleep impairment. While higher inflammatory activity was observed in postmenopausal women compared to premenopausal women in this and other investigations (e.g., [17, 56]), a large longitudinal study assessing cardiovascular disease risk markers in women before and across menopause transition stages found no differences in CRP between premenopausal, perimenopausal, and postmenopausal women [93]. It may be possible that hormonal changes occurring with menopause do not necessarily translate into increased baseline inflammation per se but may render women more vulnerable to proinflammatory conditions such as the presence of sleep disturbances. Indeed, in the current investigation, postmenopausal women with preserved sleep had similar values in CRP relative to premenopausal women, but postmenopausal women with poor sleep were more likely to have clinical elevations in CRP compared with premenopausal women with poor sleep (33.3% vs. 20%).

Higher baseline levels of CRP in older healthy women have been shown to predict the subsequent development of hypertension [94] and CV events in both hypertensive and non-hypertensive postmenopausal women [95]. Studies implicating older subjects suggest that subjective symptoms of poor sleep are associated with a greater risk for hypertension and CV disease in older women than in older men [47, 48]. While we did not show a differential association of sleep quality with inflammatory activity in men versus women in the main analyses, our findings are consistent with the hypothesis that enhanced inflammatory activation linked to poor sleep in postmenopausal women could be one of the factors linking disturbed sleep to adverse outcomes in older women. However, poor sleep and inflammatory activity were also associated in younger (premenopausal) women in this and other research. In a community sample of 43 young premenopausal women, Okun and colleagues [84] reported a cross-sectional relationship between blood levels of CRP and self-reported poor sleep quality as assessed by PSQI, after controlling for several covariates including oral contraceptive medication, menstrual phase, and education. However, only 8 subjects experienced poor sleep, leaving unclear the clinical implications of those data. Prinz [96] had also previously reported that in young adults, sleep deprivation led to metabolic, systemic, and immune changes similar to those observed with age and age-related disorders, such as CV disease.

Several factors limit the conclusions that can be drawn from this work. Given the demographics of our sample (mostly White, high functioning), the generalizability of our findings to other groups is uncertain. Moreover, the cross-sectional nature of the study prevents establishing a causal relationship between poor sleep and inflammation. Objective validation of sleep with polysomnographic or actigraphic assessment methods would have been ideal. Indeed, while women tend to report greater sleep disturbance compared to men, objective assessments tend to show women may take less time to fall asleep, sleep longer, and for a longer proportion of the night (e.g., [44]). Associations between sleep quality and inflammation may thus differ as a function of whether an objective or subjective measure of sleep is used. However, polysomnographic assessments are not routinely used in clinical practice in assessing insomnia [97] as they can be expensive, time-intensive, and inconvenient. In addition, certain aspects of sleep quality such as non-restorative sleep cannot be objectively measured [71, 85]. Moreover, in one study [44], poor sleep was associated with greater IL-6 irrespective of the sleep measure (validated questionnaire vs. polysomnographic) used in women, whereas in men, it was significant only using the PSQI. The PSQI, for its part, has been shown to be both valid and reliable in numerous populations (for example, [71, 98, 99]) and is well suited to examine qualitative aspects of sleep in addition to sleep duration and other insomnia symptoms. Nonetheless, we cannot exclude the possibility that poor sleepers in the current study may have been suffering from obstructive sleep apnea, although no participants had received a previous diagnosis of sleep apnea. The prevalence of sleep apnea in the population, particularly in healthy individuals, is considerably lower [100, 101] than the prevalence of poor sleepers observed in this study, suggesting that sleep apnea is unlikely to explain the current results. Inflammatory markers in the current study were measured only once. Repeated measurements would have permitted a better validation of inflammatory activity. A small sample size and number of participants with clinical elevations in CRP are additional limitations.

On the other hand, strengths of the study include that our population was healthy (no known illnesses or medications with the potential to impact on inflammatory processes, including hormone therapy) and well characterized in terms of socio-demographics, psychological profile, and health behaviors, which allowed us to assess the relationship between sleep and inflammatory activity independent of potential confounding. Recruitment and sampling of both men and women were performed in numbers sufficient to evaluate independent effects, with oversampling of women to permit examination of women in pre- vs. postmenopausal state. Multiple inflammatory markers were measured, increasing the confidence that can be had in results. Considering the significance of the results despite exclusion of individuals with known sleep pathologies underscores the importance of sleep to inflammatory activity.

## Perspectives and significance

In summary, poor sleep quality is associated with greater inflammation in apparently healthy individuals without a known sleep disorder, independently of sex and menopausal status. There was only limited evidence that postmenopausal women were particularly at risk. Further investigations are required to clarify the direction of effect, and the mechanisms involved in this association. Indeed, there is data to suggest that cytokine activity can regulate or modulate sleep-wake behavior [102] and that increased levels (through the administration of IL-6 for example) can contribute to sleep difficulties and increased fatigue [103]. Future studies should also longitudinally assess whether this relationship is involved in the development of chronic diseases in apparently healthy individuals, with a specific attention to differences as a function of sex and menopausal status. Screening for sleep quantity and quality and providing the necessary education or treatment for disordered sleep may be an important means through which to minimize its impact on health.

## Data Availability

The datasets used and/or analyzed during the current study are available from the corresponding author on reasonable request.
